# Long-term outcomes of osilodrostat in Cushing’s disease: LINC 3 study extension

**DOI:** 10.1530/EJE-22-0317

**Published:** 2022-08-18

**Authors:** Maria Fleseriu, John Newell-Price, Rosario Pivonello, Akira Shimatsu, Richard J Auchus, Carla Scaroni, Zhanna Belaya, Richard A Feelders, Greisa Vila, Ghislaine Houde, Rama Walia, Miguel Izquierdo, Michael Roughton, Alberto M Pedroncelli, Beverly M K Biller

**Affiliations:** 1 Pituitary Center, Departments of Medicine and Neurological Surgery, Oregon Health & Science University, Portland, Oregon, USA; 2 Department of Oncology and Metabolism, The Medical School, University of Sheffield, Sheffield, UK; 3 Dipartimento di Medicina Clinica e Chirurgia, Sezione di Endocrinologia, Università Federico II di Napoli, Naples, Italy; 4 Advanced Medical Care Center, Omi Medical Center, Kusatsu, Japan; 5 Division of Metabolism, Endocrinology and Diabetes, Departments of Internal Medicine and Pharmacology, University of Michigan, Ann Arbor, Michigan, USA; 6 Endocrinology Unit, Department of Medicine, University Hospital, Padova, Italy; 7 Department of Neuroendocrinology and Bone Disease, Endocrinology Research Centre, Moscow, Russia; 8 Department of Internal Medicine, Endocrine Section, Erasmus Medical Center, Rotterdam, The Netherlands; 9 Division of Endocrinology and Metabolism, Department of Internal Medicine III, Medical University of Vienna, Vienna, Austria; 10 Division of Endocrinology, Department of Medicine, University of Sherbrooke, Sherbrooke, Canada; 11 Department of Endocrinology, Postgraduate Institute of Medical Education and Research (PGIMER), Chandigarh, India; 12 Novartis Pharma AG, Basel, Switzerland; 13 Recordati AG, Basel, Switzerland; 14 Neuroendocrine and Pituitary Tumor Clinical Center, Massachusetts General Hospital, Boston, Massachusetts, USA

## Abstract

**Objective:**

To investigate the long-term efficacy and tolerability of osilodrostat, a potent oral 11β-hydroxylase inhibitor, for treating Cushing’s disease (CD).

**Design/methods:**

A total of 137 adults with CD and mean 24-h urinary free cortisol (mUFC) > 1.5 × upper limit of normal (ULN) received osilodrostat (starting dose 2 mg bid; maximum 30 mg bid) during the prospective, Phase III, 48-week LINC 3 (NCT02180217) core study. Patients benefiting from osilodrostat at week 48 could enter the optional extension (ending when all patients had received ≥ 72 weeks of treatment or discontinued). Efficacy and safety were assessed for all enrolled patients from the core study baseline.

**Results:**

Median osilodrostat exposure from the core study baseline to study end was 130 weeks (range 1–245) and median average dose was 7.4 mg/day (range 0.8–46.6). The reduction in mean mUFC achieved during the core was maintained during the extension and remained ≤ ULN. Of 106 patients, 86 (81%) patients who entered the extension had mUFC ≤ ULN at week 72. Improvements in cardiovascular/metabolic-related parameters, physical manifestations of hypercortisolism (fat pads, central obesity, rubor, striae, and hirsutism in females), and quality of life in the core study were also maintained or improved further during the extension. No new safety signals were reported; 15/137 (10.9%) and 12/106 (11.3%) patients discontinued for adverse events during the core and extension, respectively. Mean testosterone in females decreased towards baseline levels during the extension.

**Conclusions:**

Data from this large, multicentre trial show that long-term treatment with osilodrostat sustains cortisol normalisation alongside clinical benefits in most patients with CD and is well tolerated.

## Introduction

Patients with Cushing’s syndrome (CS) are affected by chronic exposure to excess cortisol, resulting in debilitating morbidities and an increased risk of mortality (1, 2). Cushing’s disease (CD) is the most common form of endogenous CS, caused by excess secretion of adrenocorticotropic hormone (ACTH) from a pituitary tumour, leading to the overproduction of cortisol by the adrenal glands (3). Cortisol normalisation is a key goal to reduce morbidities, including cardiovascular disease, impaired glucose metabolism, severe fatigue and weakness, emotional instability, depression, and cognitive impairments, as well as to improve physical changes (e.g. weight gain, skin thinning, striae, bruising), that may also lead to improvements in mortality and health-related quality of life (HRQoL) (1, 3, 4, 5, 6). Pituitary surgery is the first-line treatment option in most patients, requiring surgical expertise and experience to optimise outcomes (6, 7). For patients who do not achieve remission through surgery, who experience recurrent CD, who are ineligible for or refuse surgery, or who require control of cortisol levels while awaiting the effect of radiotherapy, medical therapy options are available (6, 7, 8). As patients with CD often require prolonged pharmacological treatment, evaluating the long-term efficacy and safety of drug therapies in clinical trials is essential.

Osilodrostat, a potent oral inhibitor of 11β-hydroxylase (the enzyme that catalyses the final step of cortisol synthesis), has been shown to be effective in reducing cortisol levels in patients with CD (9, 10, 11, 12). During the 48-week core phase of the prospective, multicentre, Phase III LINC 3 study (NCT02180217), osilodrostat treatment led to rapid normalisation of mean urinary free cortisol (mUFC) in most patients with CD (96% had mUFC ≤ ULN at least once during the study), alongside improvements in clinical signs of hypercortisolism, and was generally well tolerated (10). These findings were supported by a second Phase III study, LINC 4 (12). The current study reports, for the first time, results of an optional, open-ended, large open-label extension to LINC 3 conducted to gather evidence on the long-term efficacy and safety of osilodrostat.

## Methods

### Patients

As reported previously, adult patients with CD and mUFC > 1.5 times the upper limit of normal (ULN; 138 nmol/24 h or 50 μg/24 h) were enrolled in the 48-week LINC 3 core phase (10). Patients benefiting from osilodrostat treatment after 48 weeks, as assessed by the study investigator, were eligible to enter the extension phase and signed an additional written informed consent. The study was conducted in accordance with the Declaration of Helsinki, with an independent ethics committee or institutional review board at each site approving the study protocol.

### Study design

The core study design has been reported (10). In summary, all patients received open-label osilodrostat (initiated at 2 mg twice daily (bid), titrated with the aim of normalising mUFC) throughout, with the exception of the double-blind, placebo-controlled, randomised-withdrawal period (weeks 26‒34); eligible patients randomised to placebo during this period then restarted open-label osilodrostat. Patients continued to receive open-label osilodrostat during the extension, which ended after all patients completed ≥ 72 weeks of treatment or had discontinued. The study concluded when all patients were transitioned to a long-term safety follow-up study or received alternative treatment. Dose adjustments (maximum dose 30 mg bid) and interruptions were permitted throughout the extension phase to keep patients on osilodrostat; dose adjustments (including frequency of administration) other than standard dose levels were permitted based on efficacy and safety.

### Outcomes

Patients entering the extension continued assessments as follows: mUFC (mean of 2 or 3 samples; every 12 weeks, measured centrally by liquid chromatography-tandem mass spectrometry), cardiovascular- and metabolic-related parameters associated with CD (vital signs and chemistry parameters every 4 weeks, fasting plasma glucose (FPG) and HbAlc every 12 weeks), physical features (photographs from the shoulders up and of the trunk were reviewed locally by the investigator at weeks 48 and 72, with each feature (dorsal fat pad, supraclavicular fat pad, central obesity, facial rubor, proximal muscle atrophy, hirsutism (females only), ecchymoses, striae) rated subjectively on a semi-quantitative scale: 0 = absent; 1 = mild; 2 = moderate; 3 = severe), and HRQoL (Cushing’s Quality of Life (CushingQoL) questionnaire and Beck Depression Inventory II (BDI-II) conducted at weeks 48 and 72). Patients were classified as having a complete response if mUFC was ≤ ULN and a partial response if mUFC was > ULN but with a ≥ 50% reduction from baseline. Patients were categorised as uncontrolled if they achieved neither a complete nor partial response, had discontinued, or had a missing mUFC at the given time point. Other clinical and laboratory evaluations assessed and measured centrally (every 12 weeks) included total testosterone, plasma ACTH, serum morning cortisol, late-night salivary cortisol (LNSC), plasma aldosterone, DHEAS, 11-deoxycorticosterone, active renin, serum oestradiol, and oestrone. Mean percentage change in tumour volume was evaluated based on pituitary MRI (gadolinium enhanced) for patients with evaluable measurements at the core study baseline and week 72. Safety was continually assessed from the core study baseline throughout the extension for all enrolled patients by monitoring adverse events (AEs) according to Common Terminology Criteria for Adverse Events (version 4.03). Reported AEs include all data from first patient first visit to last patient last visit. AEs of special interest, anticipated according to the mechanism of action of osilodrostat, included any signs or symptoms potentially related to increases in adrenal hormone precursors, hypocortisolism, pituitary tumour enlargement, QT interval prolongation, and arrhythmogenic potential. At the end of the core study, terms to identify AEs related to pituitary tumour enlargement were revised and expanded to reflect potential pituitary tumour growth and local nerve impact. Assay details, including normal ranges, have been published (10).

### Statistical methods

Analyses were conducted after all patients had either completed at least 72 weeks or discontinued early, based on the full analysis set (all patients enrolled at the core study start who received at least one dose of osilodrostat). Safety analyses included all enrolled patients who received at least one dose of osilodrostat and had at least one valid post-baseline safety assessment. All results were analysed descriptively for all patients with an assessment at both baseline and the given visit. Two-sided 95% CIs for proportions were generated using the Clopper–Pearson exact method. No formal statistical testing was performed.

## Results

### Patient disposition

**Figure 1:**
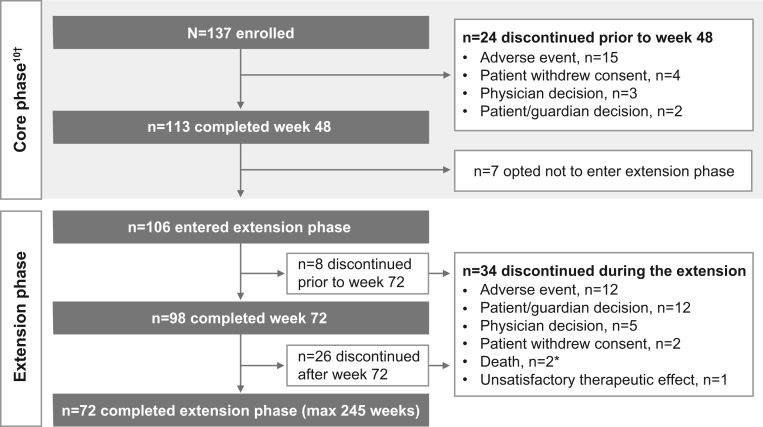
Patient disposition. *Both deaths were assessed as unrelated to osilodrostat: one case of fatal viral gastroenteritis with cardiopulmonary failure and one suicide; ^†^Please see references.

The study was conducted from 6 October 2014 to 4 December 2019. Of 137 patients enrolled in the core study, 113 completed the core phase and 106 (77.4%) opted to enter the extension phase; 98 (71.5%) completed 72 weeks of treatment (Fig. 1). Most patients had undergone previous pituitary surgery (87.6%), received prior medical therapy (74.5%), or received prior pituitary irradiation (16.1%). At the core study baseline, patients had various comorbidities, most commonly hypertension (67.9%), and physical manifestations of hypercortisolism were common (Table 1).

**Table 1:** Core study baseline characteristics of all patients (*n* = 137). Data are presented as median (range) or as *n* (%).

Characteristics	Values
Age, years	40.0 (19.0–70.0)
Sex, *n* (%)	
Male	31 (22.6)
Female	106 (77.4)
Race, *n* (%)	
Caucasian	89 (65.0)
Black	4 (2.9)
Asian	39 (28.5)
Other	5 (3.6)
Time since diagnosis, months	47.2 (2.1–286.7)
Previous pituitary surgery, *n* (%)	120 (87.6)
Previous medical therapy for CD, *n* (%)	102 (74.5)
Previous pituitary irradiation, *n* (%)	22 (16.1)
mUFC, nmol/24 h	
Mean (s.d.)	1006 (1590) (7.3 × ULN)
Median (range)	476 (36–9612) (3.5 × ULN)
Most common (≥15%) comorbidities, *n* (%)	
Hypertension	93 (67.9)
Obesity	41 (29.9)
Osteoporosis	38 (27.7)
Diabetes mellitus	30 (21.9)
Depression	27 (19.7)
Hypothyroidism	25 (18.2)
Patients categorised with physical manifestations of hypercortisolism, *n* (%)	
Dorsal fat pad	101 (73.7)
Central obesity	98 (71.5)
Supraclavicular fat pad	94 (68.6)
Facial rubor	87 (63.5)
Hirsutism (females only; *n* = 106)	62 (58.5)
Proximal muscle atrophy	71 (51.8)
Striae	67 (48.9)
Ecchymoses	53 (38.7)

mUFC was calculated as the mean of two to three samples. ULN for mUFC = 138 nmol/24 h.

### Exposure to osilodrostat

**Figure 2:**
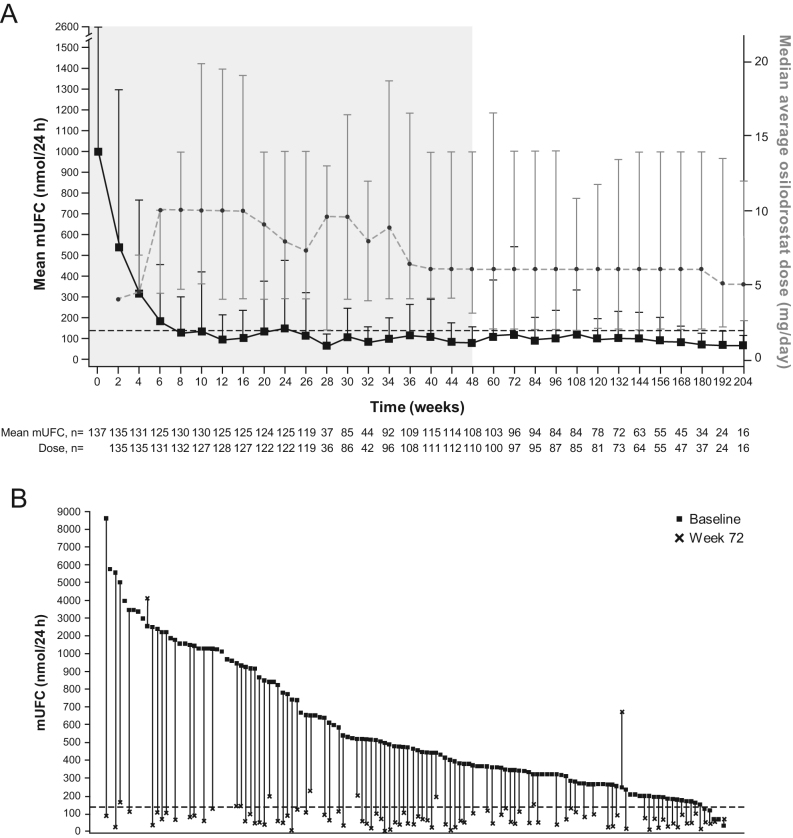
(A) Mean (+s.d.) mUFC and median (IQR) osilodrostat dose over time; (B) individual patient changes in mUFC from baseline to week 72. Shaded area in (A) indicates the core phase. The average osilodrostat total daily dose at visit X was calculated as the average of the osilodrostat total daily dose on each day between visit X and the previous visit. The reference line is the ULN of mUFC, 138 nmol/24 h.

The median duration of exposure to osilodrostat from the core study baseline to the end of the extension was 130 weeks (range 1–245). The median osilodrostat average dose from the core study start to the end of the extension was 7.4 mg/day (range 0.8–46.6, interquartile range (IQR) 3.5–13.6; Fig. 2A). The median average dose with the longest duration was 6.0 mg/day (range 0.3–60.0, IQR 2.0–14.0). From the core study baseline to the end of the extension, 124/137 (90.5%) patients received ≥ 1 dose reduction and 85/137 (62.0%) received ≥ 1 dose interruption (median duration 17.0 days (range 1‒597)).

### Long-term efficacy of osilodrostat treatment

At the end of the core period (week 48), 66.4% of patients were complete responders (*n* = 91/137; 95% CI: 57.9, 74.3). At week 60, 81.1% of patients had a complete response (*n* = 86/106; 95% CI: 72.4, 88.1). At week 72, 86/106 (81.1%; 95% CI: 72.4, 88.1) patients were complete responders (Fig. 2B). The reduction in mean mUFC observed during the core phase was maintained, and mUFC remained ≤ ULN throughout the extension (Fig. 2A).

**Figure 3:**
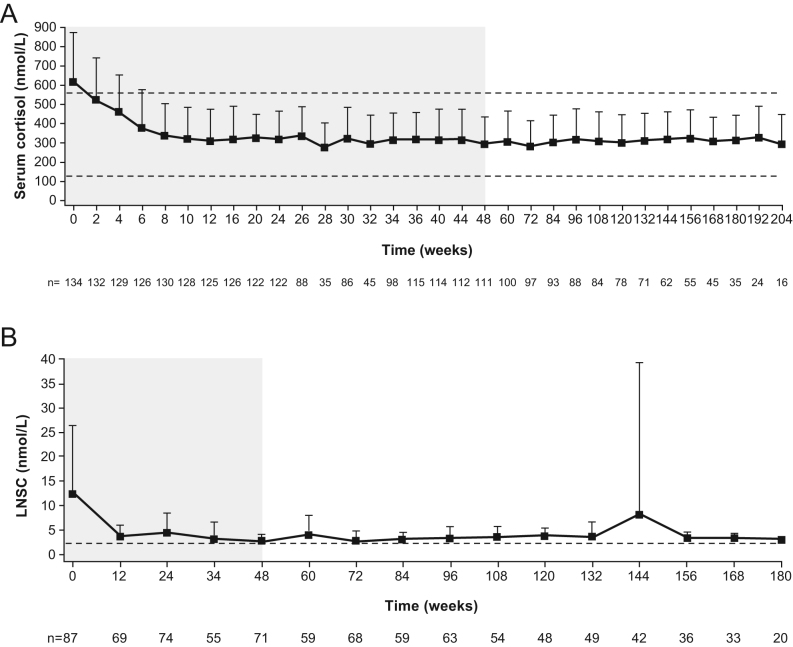
Mean (+s.d.) (A) morning serum cortisol and (B) LNSC during the study*.* Shaded areas indicate the core phase. This analysis includes scheduled visits only. *n* is the number of patients who contributed to the mean. Reference lines indicate the LLN and ULN for morning serum cortisol of 127 and 567 nmol/L, respectively, and the ULN for LNSC of 2.5 nmol/L. Different durations of follow-up are shown for serum morning cortisol and LNSC to display the longest possible duration over which data were collected.

At week 48, mean morning serum cortisol levels were within the normal range, and mean (s.d.) LNSC was 2.7 (1.6) nmol/L (ULN 2.5 nmol/L). During the extension, mean morning serum cortisol levels remained within the normal range (Fig. 3A), and LNSC remained consistently lower than baseline levels (Fig. 3B).

### Long-term changes in cardiovascular- and metabolic-related parameters and patient-reported outcomes

With longer follow-up, observed improvements in most cardiovascular- and metabolic-related parameters associated with CD at the end of the core study (10) were maintained or further improved (Table 2). Increases from baseline in mean CushingQoL score (indicating improvement) were observed at all post-baseline visits reaching the distribution-based minimum important difference (MID; 10.1-point change from baseline) (13) at all time points (Table 2). Improvements in total CushingQoL score were reflective of beneficial changes in both the physical problems and psychosocial issues subscores. Decreases in total BDI-II score (indicating improvement) were also observed at all post-baseline visits and reached MID values (17.5% reduction from baseline) (14) at all time points (Table 2).

**Table 2:** Mean change from the core study baseline in cardiovascular- and metabolic-related parameters and patient-reported outcomes. Data are presented as mean (s.d.).

Parameter	Core study baseline (10)	Change from core study baseline at	
		Week 48	Week 72
Weight, kg	80.8 (22.4)	−3.8 (5.7)	−4.7 (6.6)
BMI, kg/m^2^	30.3 (7.8)	−1.4 (2.2)	−1.8 (2.5)
Waist circumference, cm	103.5 (19.3)	−4.7 (7.8)	−6.2 (8.5)
Systolic blood pressure, mmHg	132.2 (15.1)	−9.8 (15.5)	−10.1 (18.1)
Diastolic blood pressure, mmHg	85.3 (10.6)	−6.3 (11.1)	−5.8 (11.3)
FPG, mmol/L	5.5 (1.7)	−0.5 (1.3)	−0.3 (1.1)
HbA1c, %	6.0 (1.0)	−0.4 (0.7)	−0.4 (0.6)
Total cholesterol, mmol/L	5.3 (1.2)	−0.5 (0.9)	−0.4 (0.9)
Triglycerides, mmol/L	1.5 (1.3)	−0.1 (0.9)	−0.1 (0.6)
Cushing QoL total score	42.2 (19.1)	+14.0 (16.8)	+15.1 (19.4)
Physical problems subscore	38.9 (23.7)	+18.3 (22.0)	+19.3 (22.9)
Psychosocial issues subscore	43.3 (20.4)	+12.7 (17.4)	+13.6 (20.1)
BDI-II score	16.8 (10.6)	−5.8 (9.5)	−6.7 (10.7)

Normal ranges: plasma glucose, 3.9–5.5 mmol/L; HbA1c, ≤6.4%; total cholesterol, ≤4.4 mmol/L in patients aged <19 years and ≤5.2 mmol/L in patients aged ≥20 years; triglycerides, ≤2.2 mmol/L. Increases in CushingQoL and decreases in BDI-II scores indicate improvements.

### Long-term changes in physical manifestations of hypercortisolism

**Figure 4:**
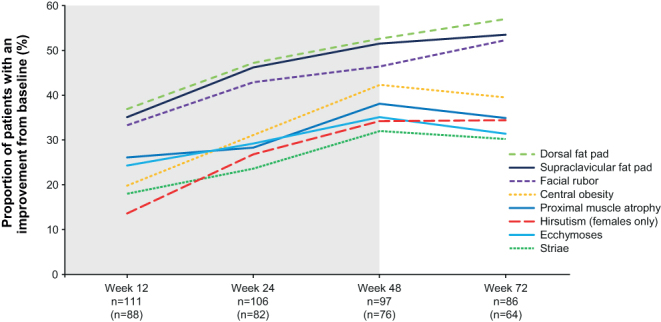
Proportion of patients with an improvement from baseline in physical manifestations of hypercortisolism over time. Shaded area indicates the core phase. An improvement was defined as the symptom score being lower (i.e. less severe) than at baseline. The denominator for the percentage is the number of patients in the full analysis set (all enrolled patients who received at least one dose of osilodrostat; shown in brackets for female patients assessed for hirsutism), with data available at both baseline and the given visit. *n* = 105 at week 24 for facial rubor.

Improvements in physician-rated severity scores for assessed physical manifestations of hypercortisolism were evident within 12 weeks of osilodrostat treatment (Fig. 4); the proportion of patients rated with an improvement was maintained or increased with longer follow-up. This included hirsutism in female patients, whereby 86.4% of patients had an improved or stable severity score at week 72; stabilisation or improvements occurred in patients with both normal and elevated testosterone levels during the study (Supplementary Table 1, see section on supplementary materials given at the end of this article). At week 72, improved scores were observed in patients rated as having mild, moderate, or severe physical manifestations at baseline; few patients experienced worsening (Supplementary Fig. 1).

### Adverse events

Throughout the study (median exposure to osilodrostat 130 (range 1‒245) weeks), the most common AEs regardless of study-drug relationship were nausea (*n* = 62, 45.3%), headache (*n* = 50, 36.5%), and fatigue (*n* = 45, 32.8%) (Table 3). In total, 15/137 (10.9%) patients discontinued the study because of an AE during the core phase, and 12/106 (11.3%) discontinued because of an AE during the extension.

**Table 3:** Most common AEs (> 10%) by preferred term throughout the core and extension regardless of study-drug relationship in all patients (*n* = 137). Data are presented as *n* (%).

	All grades	Grade 3/4
Any AE	137 (100)	83 (60.6)
AEs leading to discontinuation	25 (18.2)	17 (12.4)
AEs requiring dose adjustment/interruption	110 (80.3)	42 (30.7)
AEs requiring additional therapy	132 (96.4)	61 (44.5)
Most common AEs		
Nausea	62 (45.3)	3 (2.2)
Headache	50 (36.5)	6 (4.4)
Fatigue	45 (32.8)	3 (2.2)
Adrenal insufficiency*	40 (29.2)	6 (4.4)
Vomiting	34 (24.8)	5 (3.6)
Nasopharyngitis	33 (24.1)	1 (0.7)
Arthralgia	29 (21.2)	3 (2.2)
Back pain	29 (21.2)	0
Increased blood corticotropin	28 (20.4)	1 (0.7)
Glucocorticoid deficiency^†^	28 (20.4)	5 (3.6)
Asthenia	27 (19.7)	1 (0.7)
Diarrhoea	27 (19.7)	1 (0.7)
Dizziness	26 (19.0)	0
Influenza	26 (19.0)	1 (0.7)
Urinary tract infection	25 (18.2)	3 (2.2)
Hypertension	24 (17.5)	16 (11.7)
Decreased appetite	22 (16.1)	0
Peripheral oedema	22 (16.1)	0
Pyrexia	21 (15.3)	0
Rash	21 (15.3)	1 (0.7)
Cough	20 (14.6)	2 (1.5)
Myalgia	20 (14.6)	0
Abdominal pain	18 (13.1)	4 (2.9)
Abnormal hormone level	18 (13.1)	0
Hypokalaemia	18 (13.1)	6 (4.4)
Increased blood testosterone	16 (11.7)	0
Anaemia	15 (10.9)	2 (1.5)
Dyspepsia	15 (10.9)	0
Oropharyngeal pain	14 (10.2)	1 (0.7)
Pain in extremity	14 (10.2)	3 (2.2)
Upper respiratory tract infection	14 (10.2)	0

A patient with multiple severity grades for an AE is only counted under the maximum grade. Terms based on phrasing by the investigator: *Adrenal insufficiency includes ‘relative adrenal insufficiency’, ‘adrenocortical insufficiency’, ‘hypoadrenocorticism’, ‘suspected hypoadrenalism’, ‘mild adrenal insufficiency’, and ‘adrenal deficiency’; ^†^Glucocorticoid deficiency includes ‘hypocortisolism’, ‘symptoms of hypocortisolism’, ‘relative hypocortisolism’, ‘suspicion of hypocortisolism’, ‘asymptomatic/symptomatic hypocortisolism’, and ‘subjective symptoms of hypocortisolism’.

**Figure 5:**
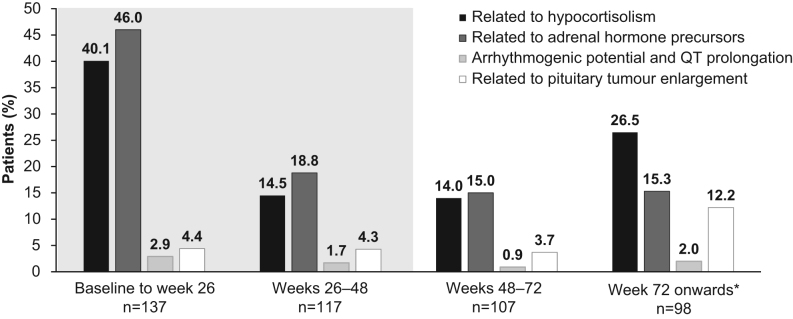
Occurrence of AEs of special interest by time interval. Shaded area indicates the core phase. *n* is the number of patients with ≥1 scheduled visit or AE during the time interval. *Maximum exposure 245 weeks.

AEs of special interest were reported regardless of study-drug relationship. Hypocortisolism-related AEs (including investigator-assessed adrenal insufficiency and glucocorticoid deficiency) occurred in 74/137 (54.0%) patients and were most common during the first 26 weeks of the study (Fig. 5); 4 patients discontinued as a result during the core phase and only 1 during the extension. In total, 67 (48.9%) patients with hypocortisolism-related AEs were managed with temporary interruption of osilodrostat, 41 (60.3%) patients received a dose reduction, and 31 (22.6%) patients received glucocorticoid therapy. AEs related to adrenal hormone precursors occurred in 80/137 (58.4%) patients and were also less frequent in the extension than in the core study; two patients discontinued as a result in the core study and none during the extension. The most common AEs related to adrenal hormone precursors (> 10% of patients throughout the study; Table 3) were hypertension (*n* = 24, 17.5%; 19 events during the core, 13 during the extension), peripheral oedema (*n* = 22, 16.1%; 24 events during the core, 4 during the extension), hypokalaemia (*n* = 18, 13.1%; 17 events during the core, 5 during the extension), and increased blood testosterone (*n* = 16, 11.7%; 17 events during the core, 1 during the extension). The occurrence of AEs potentially related to arrhythmogenic potential and QT prolongation remained infrequent throughout the study. An increase in AEs related to pituitary tumour enlargement was noted from week 72 onwards (maximum exposure 245 weeks). Overall, 13 patients with AEs related to pituitary tumour enlargement discontinued treatment as a result: 8 patients during the core phase and 5 during the extension. Reported AEs related to pituitary tumour enlargement were pituitary tumour benign (*n* = 12, 8.8%), pituitary tumour (*n* = 7, 5.1%), diplopia (*n* = 5, 3.6%), sixth nerve paralysis (*n* = 3, 2.2%), pituitary infarction (*n* = 1, 0.7%), and tumour invasion (*n* = 1, 0.7%). The median change in tumour volume as assessed by MRI from the core study baseline to week 72 was 1.0 mm^3^ (range  74.7 to 1268.5). Overall, 16 (29.6%) patients with a measurable tumour at baseline and at least 1 post-baseline assessment had a ≥ 20% decrease in tumour volume at week 72 (median treatment duration 144 weeks), and 21 (38.9%) had a ≥ 20% increase (median treatment duration 120 weeks). Stable doses of concomitant medications (excluding those for hypercortisolism) were permitted throughout the study. All patients received concomitant medications, most commonly (initiated during the study in > 15% of patients) paracetamol (48.9%), spironolactone (24.1%), hydrocortisone (23.4%), ibuprofen (23.4%), metformin (15.3%), amoxicillin (15.3%), levothyroxine sodium (24.8%), cholecalciferol (29.9%), and potassium chloride (19.0%). Of patients taking hypertensive or antidiabetic medications at baseline, 40% (*n* = 34/85) and 23% (*n* = 10/43) of patients, respectively, increased their dose or number of concomitant medications while 40% (*n* = 34/85) and 49% (*n* = 21/43) of patients, respectively, stopped or reduced the dose by the end of the core phase (week 48) (15).

### Long-term changes in mean hormone levels

**Figure 6:**
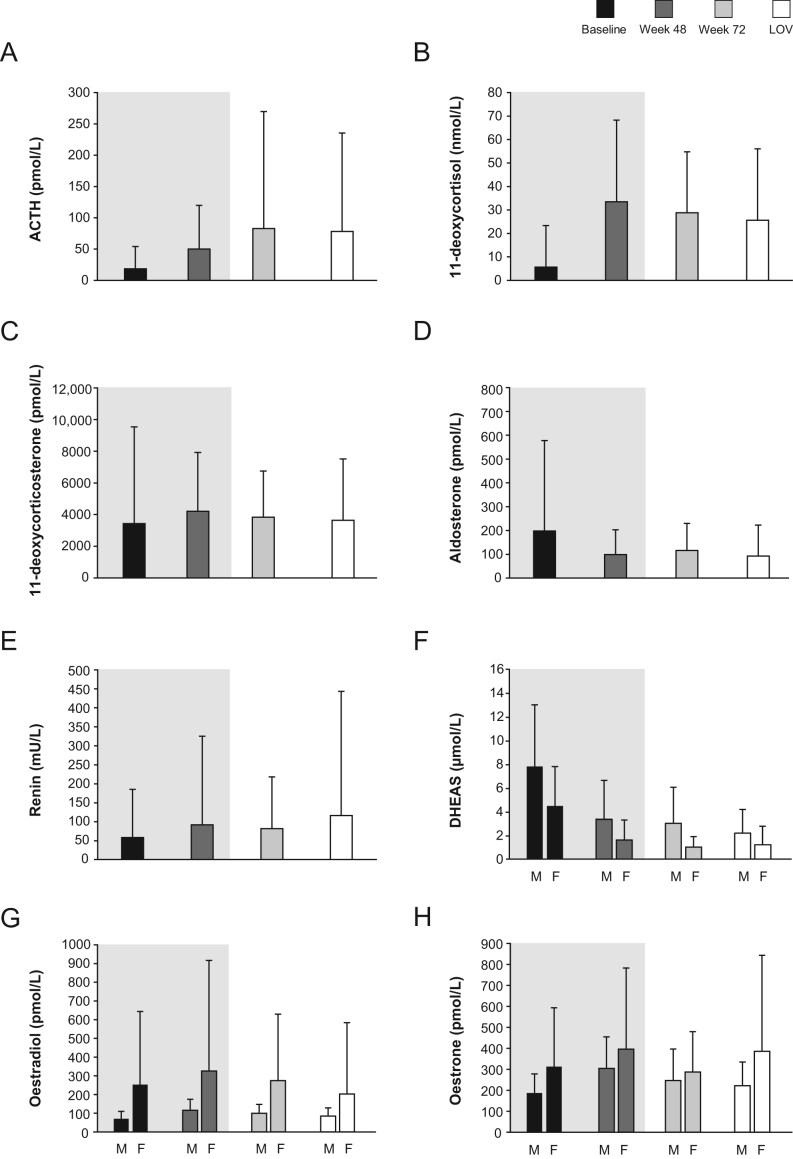
Mean (+s.d.) hormone levels up to the last observed value in the extension phase for (A) ACTH, (B) 11-deoxycortisol, (C) 11-deoxycorticosterone, (D) aldosterone, (E) renin, (F) DHEAS, (G) oestradiol, and (H) oestrone. ACTH normal range: 1.6–11.1 pmol/L in males and 1.1–6.0 pmol/L in females; 11-deoxycortisol ULN, 3.9 nmol/L in males and 3.1 nmol/L in females, or lower depending on age; 11-deoxycorticosterone ULN, 455 pmol/L in males and 696 pmol/L in females (mid-cycle); plasma aldosterone ULN, 777 pmol/L (upright, 08:00‒10:00); DHEAS ULN, 18.8 µmol/L in males and 10.6 μmol/L in females, or lower depending on age; renin ULN, 46.1 mU/L; serum oestradiol ULN, 106 pmol/L in males and 2797 pmol/L in females (mid-cycle); oestrone ULN, 255 pmol/L in males and 991 pmol/L in females (mid-cycle). F, female; M, male.

Mean (s.d.) ACTH levels were increased at the end of the core study (50.0 (35.5) pmol/L at week 48 (normal range 1.6–11.1 in males and 1.1–6.0 in females)) then stabilised during the extension phase; mean ACTH was 82.6 (187.2) pmol/L at week 72 and 78.1 (157.2) pmol/L at the last observed value (LOV; Fig. 6A). Following an increase in mean (s.d.) 11-deoxycortisol levels during the core phase (33.5 (34.8) nmol/L at week 48 (ULN 3.9 in males and 3.1 in females, or lower depending on age)), mean levels decreased to 25.6 (30.5) nmol/L at the LOV (Fig. 6B). Mean 11-deoxycorticosterone levels stabilised with longer follow-up, with no apparent changes at the LOV compared with the end of the core period (ULN 455 pmol/L in males and 696 pmol/L in females (mid-cycle); Fig. 6C). Reductions observed in plasma aldosterone (ULN 777 pmol/L (upright, 08:00‒10:00); Fig. 6D) and DHEAS (ULN 18.8 µmol/L in males and 10.6 μmol/L in females, or lower depending on age; Fig. 6F) during the core study stabilised during the extension, and increases in renin (ULN 46.1 mU/L; Fig. 6E), serum oestradiol (ULN 106 pmol/L in males and 2797 pmol/L in females (mid-cycle); Fig. 6G), and oestrone (ULN 255 pmol/L in males and 991 pmol/L in females (mid-cycle); Fig. 6H) observed during the core study also stabilised during the extension.

**Figure 7:**
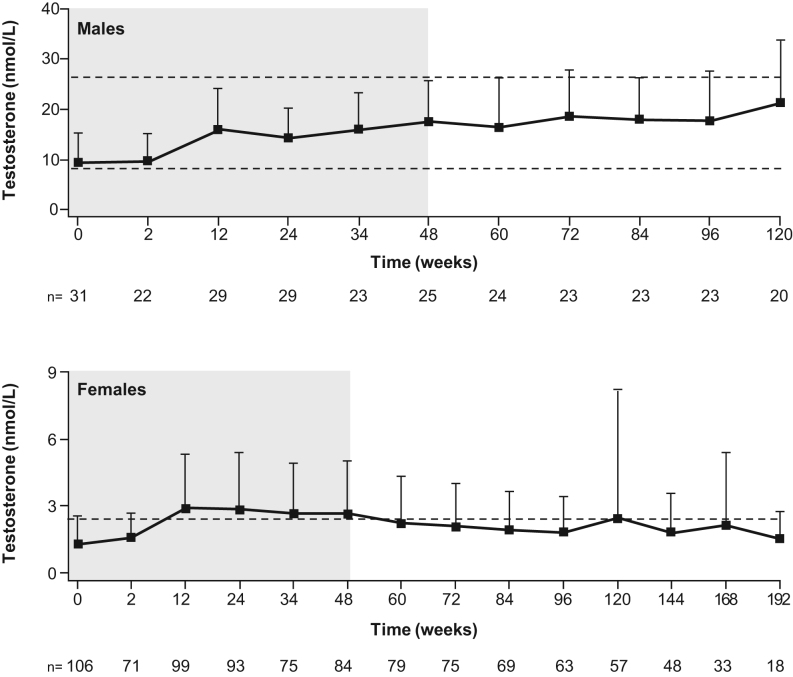
Mean (+s.d.) testosterone levels in males and females. Testosterone reference range: 8.4–28.7 nmol/L (males), 0.7–2.6 nmol/L (females). Shaded area indicates the core phase. Different durations of follow-up are shown to display the longest possible duration over which data were collected.

No substantial changes in mean testosterone levels were observed in male patients compared with levels reported at the end of the core phase (Fig. 7). In female patients, mean (s.d.) testosterone levels increased from 1.3 (1.2) nmol/L at baseline to 2.6 (2.4) nmol/L at the end of the core phase, then decreased to within the normal range (0.7‒2.6 nmol/L for females) throughout the extension phase; 2.1 (1.9) nmol/L at week 72 and 1.8 (2.0) nmol/L at the LOV. During the core phase, 12 female patients had an AE of hirsutism; all were grade 1–2, and none resulted in study discontinuation (10). No new hirsutism AEs were reported during the extension, and no patient discontinued because of AEs related to increased blood testosterone.

## Discussion

To our knowledge, this is the largest prospective trial of an adrenal steroidogenesis inhibitor with long-term follow-up in patients with CD. The optional extension phase to LINC 3 reported here confirmed that the rapid control of mUFC in the core phase (10) was sustained over time, with a median exposure of 130 weeks (maximum 245 weeks). At week 72, most patients (88.7%) were classified as mUFC responders, with 81.1% classified as complete responders. This was accompanied by sustained improvements in morning serum cortisol and LNSC, as well as cardiovascular/metabolic-related parameters, physical manifestations of hypercortisolism, and HRQoL. With longer follow-up, no new or unexpected safety findings occurred.

The demographic and disease characteristics of enrolled patients were representative of a CD population with a severe burden of disease. Most patients had physical manifestations of hypercortisolism, as well as hypertension, with considerably elevated mUFC despite prior medical therapy and/or pituitary surgery. The optional retention of 106 patients in the extension phase supports the benefits of osilodrostat in this difficult-to-treat patient population. The median average osilodrostat dose stabilised during the extension, indicating that osilodrostat treatment provides a sustained response without the need for uptitration over time. However, individualised monitoring of patients receiving medical therapy for CD in clinical practice is recommended given the possibility of hypocortisolism-related AEs (6).

The maintained reductions in mUFC and LNSC with long-term treatment were accompanied by sustained improvements in cardiovascular/metabolic-related parameters. These findings are clinically important, considering that many patients with CD are hypertensive, are often overweight or obese, have disturbances in glucose and lipid metabolism (3), and have many comorbidities as described earlier. Furthermore, there were also continued numerical improvements in weight and waist circumference with longer follow-up. Improvements in physician-rated severity scores for physical manifestations of hypercortisolism also occurred soon after osilodrostat initiation and were sustained or continued to improve throughout 72 weeks in many patients, including hirsutism in female patients. The early and progressive improvements seen in female patients for hirsutism severity score are reassuring, given the potential for increases in testosterone levels following the initiation of osilodrostat. Notably, testosterone levels tended to return to baseline levels with longer follow-up, and no new AEs related to increased blood testosterone were reported during the extension phase. Clinically meaningful improvements in QoL indicators were sustained throughout the extension. Taken together, these improvements highlight considerable long-term benefits to patients of controlling hypercortisolism with osilodrostat. Exploring the relationship between normalisation of mUFC and/or LNSC and improvements in clinical parameters and QoL is of interest, as data indicate better outcomes in patients with normalisation of both parameters (16, 17).

Osilodrostat was generally well tolerated, with no new or unexpected safety findings with long-term treatment. The most common AEs included nausea and fatigue, a proportion of which may have been related to hypocortisolism-related events, though not categorised as such by the investigators and glucocorticoid withdrawal could not be excluded. The proportion of patients discontinuing treatment because of AEs in the extension continued to be low, similar to the core study. AEs of special interest, including AEs related to hypocortisolism and adrenal hormone precursors, mostly occurred during the first 26 weeks (dose-titration period) and generally decreased thereafter. Hypocortisolism-related AEs were not associated with mUFC levels or specific osilodrostat dose at the time of the AE (18). As noted in the recent Pituitary Society guidelines, when treatment with steroidogenesis inhibitors is dose titrated to achieve cortisol normalisation, there is a risk of adrenal insufficiency with overtreatment (6). Indeed, the reduction in hypocortisolism-related AEs after week 26 was accompanied by a lower median osilodrostat dose. Gradual dose titration in clinical practice alongside the education of patients to recognise potential signs of hypocortisolism may help reduce the risk of hypocortisolism-related AEs (19). In this clinical trial, very few patients discontinued because of hypocortisolism-related AEs (3.6% overall; *n* = 4 during the core and *n* = 1 during the extension), indicating that they are mild and manageable.

Levels of adrenal hormone precursors increased during osilodrostat treatment in the core phase; potential associated AEs (such as hypertension, oedema, and hypokalaemia) were largely treated through temporary drug interruptions or additional medications, with few patients discontinuing treatment. The reduced occurrence of AEs related to adrenal hormone precursors after week 26 is consistent with data indicating minimal changes in adrenal hormone precursors with longer follow-up. During the extension phase, there was a trend for levels of ACTH, renin, plasma aldosterone, DHEAS, 11-deoxycortisol, and 11-deoxycorticosterone (cortisol and aldosterone precursors, respectively) to either stabilise or decrease. In male patients, levels of oestradiol, oestrone, and testosterone remained stable throughout the study. In female patients, oestrone levels remained stable, and oestradiol and testosterone levels tended to return to baseline levels with longer follow-up. Reductions in testosterone levels most likely accounted for the lack of investigator-reported AEs related to hirsutism or increased blood testosterone during the extension phase. As such, this study indicates that osilodrostat can be considered for both male and female patients.

The increase in AEs related to pituitary tumours from week 72 onwards (reported in < 5% of patients up to week 72, then in 12.2% of patients from week 72 onwards) was not unexpected given the length of follow-up and the underlying recurrent nature of the disease (6), although we also cannot rule out a potential contributory effect of the medication. Furthermore, after the primary endpoint analysis at the end of the core period of the study (at which time only three patients had relevant AEs, reported as diplopia), the terms chosen to identify AEs related to pituitary tumour enlargement were revised and expanded. With the expanded search terms, 19 additional patients were identified with AEs related to pituitary tumour enlargement, resulting in a total of 22 patients throughout the study. However, in patients with a measurable tumour by MRI at baseline, median pituitary tumour volume remained relatively stable with long-term follow-up. It is reassuring that there was no evidence of rapid tumour growth during the study, and few patients discontinued the study because of AEs related to pituitary tumour enlargement; however, all patients on adrenal-blocking medication should have regular imaging at planned intervals and sooner if symptoms occur (6, 20).

This study adds long-term data to the robust evidence for osilodrostat, supporting its efficacy and safety in patients with CD (9, 10, 11, 12). Other adrenal steroidogenesis inhibitors have been used for decades in clinical practice based on pragmatic experience, but long-term clinical trial data are currently limited. Retrospective data for ketoconazole with ~2 years of patient follow-up indicated reduced mUFC with an acceptable safety profile (21). Although ~65% of patients initially had normalised mUFC, 15–25% experienced escape from response (21). In such cases, increased doses may be required (6). Levoketoconazole, a stereoisomer of ketoconazole, has recently been FDA approved (22, 23, 24). In the pivotal Phase III prospective trial, mUFC normalisation was achieved in 31% of patients at 6 months (22). Risks of drug‒drug interactions, hepatotoxicity, and QT prolongation require careful monitoring prior to and during treatment. Early findings from the ongoing prospective PROMPT study, evaluating metyrapone in patients with CS, indicated normalisation of mUFC in 47% of patients at week 12 (25), supporting outcomes from retrospective (26) and observational studies (27). However, metyrapone generally requires three or four doses a day, which can affect compliance in some patients (4). In the absence of head-to-head comparative clinical trial data, medical therapy choice is guided by efficacy, risk of AEs and drug–drug interactions, cost, availability, and other factors specific to individual patients (1, 6, 28, 29).

We acknowledge the limitations of this study, including the potential for selection bias for patients who experienced the greatest benefit in the core study. LINC 3 was, however, a large prospective study, and most patients chose to continue into the extension after providing additional informed consent. We included data from the full patient population enrolled at the study baseline to avoid selection bias as far as possible. We also cannot exclude the possibility that reduced mUFC could be attributed to the delayed effects of radiation in a small number of patients. As such, an evaluation of long-term response rates in clinical practice is needed to support findings from clinical trials.

In summary, durable cortisol normalisation was observed in the extension phase of the prospective LINC 3 study evaluating osilodrostat. To our knowledge, this was also the first long-term study analysing changes in physical manifestations of hypercortisolism during medical therapy. We have shown here that sustained biochemical control was accompanied by enduring improvements in both clinical signs/features of hypercortisolism and patient-reported outcomes. Patients should be regularly monitored and osilodrostat dose titrated as necessary, alongside adjustment of concomitant medications, to optimise outcomes. Taken together, these findings support osilodrostat as an effective and well-tolerated long-term treatment option for patients with CD.

## Supplementary Material

Supplementary Material

## Declaration of interests

M F reports grants to her university and occasional scientific consulting fees from Recordati Rare Diseases and Strongbridge and consulting fees from HRA Pharma and Sparrow; she served as a member of the LINC 3 steering committee. M F is also a Deputy Editor of *EJE*. M F was not involved in the review or editorial process for this paper on which she is listed as an author. J N P reports grants and consultancy payments to his university from Crinetics, Diurnal, HRA Pharma, and Recordati Rare Diseases. R P has received research support to Università Federico II di Napoli as a principal investigator for clinical trials from Novartis Pharma, Recordati, Strongbridge Biopharma, Corcept Therapeutics, HRA Pharma, Shire, Takeda, Neurocrine Biosciences, Camurus AB, and Pfizer; has received research support to Università Federico II di Napoli from Pfizer, Ipsen, Novartis Pharma, Strongbridge Biopharma, Merk Serono, and Ibsa; and received occasional consulting honoraria from Novartis Pharma, Recordati, Strongbridge Biopharma, HRA Pharma, Crinetics Pharmaceuticals, Corcept Therapeutics, Pfizer, and Bresmed Health Solutions. A S reports serving as a speaker and consultant to Recordati and as a member of the LINC 3 steering committee. R J A reports grants and personal fees from Spruce Biosciences, Neurocrine Biosciences, Corcept Therapeutics, and Novartis and personal fees from Janssen Pharmaceuticals, Crinetics Pharmaceuticals, OMass Therapeutics, Quest Diagnostics, Adrenas Therapeutics, and PhaseBio. C S reports occasional consulting honoraria from HRA, Novartis, Recordati, Pfizer, and Sandoz. Z B has no conflicts of interest to disclose. R A F reports research grants from Strongbridge and Corcept Therapeutics and consultancy fees from Recordati Rare Diseases and Corcept Therapeutics. G V reports serving as a speaker and consultant for Novartis, HRA Pharma, and Recordati and as a research investigator for Novartis, Corcept, and Recordati. G H reports serving as a consultant for Novartis. R W has no conflicts of interest to disclose. M I and M R are employees of Novartis. A M P and A P are employees of Recordati. B M K B reports research grants to her institution from Novartis, Strongbridge, and Millendo and occasional consulting honoraria from HRA Pharma, Recordati Rare Diseases, and Sparrow; she served as a member of the LINC 3 steering committee.

## Funding

This study was funded by Novartis Pharma AG; however, as of 12 July 2019, osilodrostat is an asset of Recordati. Financial support for medical editorial assistance was provided by Recordati.
