# Supplementary material for: Long-term outcomes of osilodrostat in Cushing’s disease: LINC 3 study extension
Source: Eur J Endocrinol. 2022 Aug 18;187(4):531–41. doi: 10.1530/EJE-22-0317 (PMC9513654; doi:10.1530/EJE-22-0317)

Long-term outcomes of osilodrostat in Cushing's disease: LINC 3 study extension

Supplementary information

Supplementary Table 1. Summary of female patients with improved, worsened, or stable hirsutism score ratings over time according to testosterone levels

Testosterone levels	Hirsutism score	Time (weeks)			
		12	24	48	72
**Normal**	Improved	n=9/38 (23.7%)	n=16/38 (42.1%)	n=16/34 (47.1%)	n=17/38 (44.7%)
	Worsened	n=2/38 (5.3%)	n=4/38 (10.5%)	n=5/34 (14.7%)	n=5/38 (13.2%)
	Stable	n=27/38 (71.1%)	n=18/38 (47.4%)	n=13/34 (38.2%)	n=16/38 (42.1%)
**>ULN**	Improved	n=3/48 (6.3%)	n=5/42 (11.9%)	n=9/41 (22.0%)	n=6/28 (21.4%)
	Worsened	n=4/48 (8.3%)	n=6/42 (14.3%)	n=8/41 (19.5%)	n=4/28 (14.3%)
	Stable	n=41/48 (85.4%)	n=31/42 (73.8%)	n=24/41 (58.5%)	n=18/28 (64.3%)

ULN, upper limit of normal

Supplementary Figure 1. Shift in severity score ratings for physical manifestations of hypercortisolism from baseline to week 72

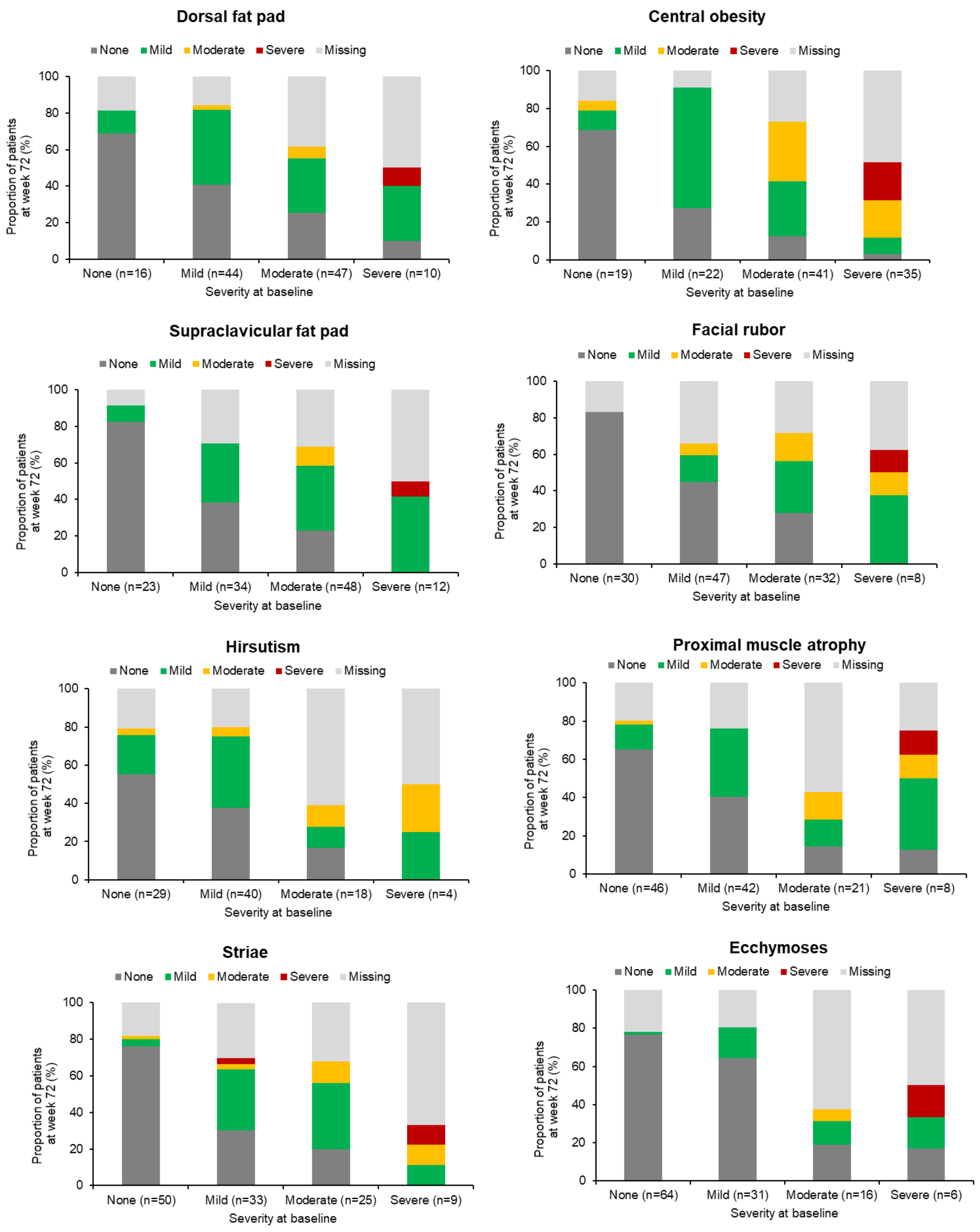

Supplement: Supplementary Material [file supplementary_material.pdf]
